# Rationale for Targeting CD6 as a Treatment for Autoimmune Diseases

**DOI:** 10.1155/2010/130646

**Published:** 2011-02-10

**Authors:** Ruby Alonso-Ramirez, Séverine Loisel, Caroline Buors, Jacques-Olivier Pers, Enrique Montero, Pierre Youinou, Yves Renaudineau

**Affiliations:** ^1^EA2216 Immunology and Pathology and IFR 148 ScInBioS, European University of Brittany, BP 824, 29609 Brest, France; ^2^Experimental Immunotherapy Department, Center for Molecular Immunology, P.O. Box 16040, 11600 La Havana, Cuba; ^3^Laboratory of Immunology, CHU Brest, Brest University Medical School Hospital, BP 824, 29609 Brest, France

## Abstract

CD6 is a 105–130 kDa surface glycoprotein expressed on the majority of T cells and a subset of B cells. The human *cd6* gene maps to chromosome 11, and the expression of its protein product is tightly regulated. CD6 mediates cellular adhesion migration across the endothelial and epithelial cells. In addition, it participates in the antigen presentation by B cells and the subsequent proliferation of T cells. CD6 may bind in *trans* to surface glycoproteins (such as ALCAM and 3A11), or to microbial lipopolysaccharides, and may bind in *cis* to endogenous ligands (such as CD3 and CD5), and thereby deliver a costimulatory signal. Transinteractions are reinforced during autoimmune diseases (e.g., rheumatoid arthritis (RA), Sjögren's syndrome, and multiple sclerosis) and some cancers. Based on experimental data and on clinical results in RA and psoriasis, we believe that the recent humanized anti-CD6-specific mAb T1h may act as a regulator of the immunological response in addition to its function as an anti-T- and -B cell agent.

## 1. Introduction

The past few years have witnessed increasing interest in the possibility that B lymphocytes are cast with a leading role in the play of autoimmunity. Treatment with B cell-depleting agents has thus become the logics. Among these is the anti-CD6 antibodies (Abs), because CD6 is harboured, not only by all T cells, but also by some B cells. The earliest successes of anti-CD6 Abs in autoimmune diseases [1–3] were obtained in the treatment of rheumatoid arthritis (RA), psoriasis, and multiple sclerosis (MS) (Table 1). In spite of such promising results, the investigators had to give up [4, 5] due to the murine origin of their monoclonal Abs (mAbs). There was thus a need for humanized anti-CD6 mAb. This task was accomplished by mutagenesis in 2003 using the murine anti-CD6 single scavenger receptor cystein-rich (SRCR) domain 1, ior T1 [6, 7]. The resulting humanized anti-CD6 mAb was termed T1h, and evaluated in RA and psoriasis. The preliminary results in active RA [8] revealed that the combination of T1h, with methotrexate resulted in a long-term remission with a significative reduction of swollen and tender joints. The question, therefore, arises as to how the anti-CD6 T1h mAb work? First, one might argue that anti-CD6 Ab should target the T cells, as well. Were that to be the case, a T-cell depletion should be elicited such was not the case. Second, it may block interactions between CD6 and the activated leukocyte cell adhesion molecule (ALCAM). However, *in vivo* experiments and *in vitro* competition assays have cast doubt on this possibility. By self-definition, it is important to define the rationale for using anti-CD6 mAb in autoimmune diseases. The present review summarizes our knowledge regarding CD6, its functions, and the different anti-CD6 mAbs that have been developed and tested.

## 2. CD6 from the Gene to the Protein

### 2.1. cd6 Gene

**Figure 1:**
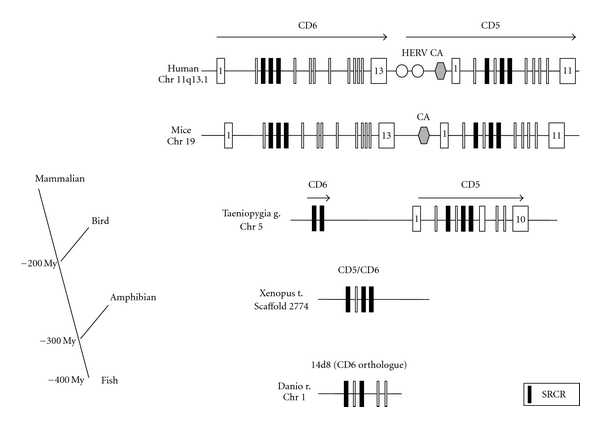
CD6 and CD5 genes map to contiguous regions in mammalian and birds and are supposed to have arisen for implication of a common ancestral gene. CD6 is orthologue to the amphibian protein CD5/CD6 and the fish protein 14d8. Data has been taken from the genome database (http://www.ensembl.org/) and from previously published results [11, 13]. Black boxes represent the exons encoding the extracellular SRCR domains. First and last exons are depicted when known. Legend: HERV human endogenous retrovirus; CA: microsatellite repeat, My: million year.

The gene for *cd6*, a T-cell differentiation antigen (Ag) maps to chromosome 11 at position 11q13.1, spans more than 25-kb adjacently, and adjoins the *cd5* gene within 200-kb telomeric to *cd20* in human [9]. In mice and chicken, the *cd6* gene is present on chromosome 19 and 5, respectively, [10, 11]. Phylogenetic analysis supports the concept that *cd6* and *cd5* have emerged from duplication of a common ancestor, probably before the separation of mammalian/birds and amphibian 200–300 million years ago (Figure 1). Interestingly, in humans, but not in mice, an endogenous retrovirus type E is inserted between *cd6* and *cd5* genes, and serves as an alternative gene promoter for *cd5.* The influence on *cd6* is unknown [12, 13].

A minimal *cd6* promoter has been recently described at positions −506/−146 from the start codon ATG (+1) [14]. Mutational analysis of the promoter and characterization of related transcription factors (TFs) have identified Ets-1 and RUNX1/3 as *cd6* regulators. Likewise, *cd5* gene is positively regulated by Ets-1 and NFAT, and negatively regulated by E47 [15–17]. The inhibitory effect of E47 on the expression of CD6 has never been described, even though the *cd6* promoter contains an E-box-binding domain at position −273. In addition, *cd6* promoter is regulated at the epigenetic level through methylation of its 15 CpG motifs between positions −300/+1. As observed by treating cloned human T cells with the DNA methylation inhibitor 5-azacytidine [18]. 

**Figure 2:**
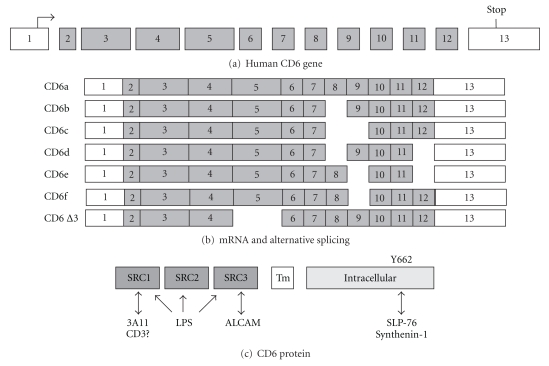
Human CD6 transcripts. (a) Diagram of the human CD6 gene exons which are depicted as boxes numbered from 1 to 13. (b) Representation of the different CD6 isoforms. (c) Scheme of the CD6 protein. The localization of the different binding domains is indicated.

RNase protection assay and 5′RACE RT-PCR show multiple transcriptional start sites in accordance with the absence of TATA box in the 5′ flanking region of *cd*6 [14]. In humans, exon 1 contains the start codon and exon 13 contain the stop codon. In between, a nucleotide-long-related open reading frame corresponds to a 668-amino acid (aa) protein. Exons 1 and 2 encode the 24-aa signal sequence, exons 3 through 5 encode each a 101/117-aa SRCR domain, exon 6 encodes a 22-aa spacer that separates the SRCR-D3 from the exon 7-encoded 26-aa transmembrane domain, while exons 8 through 13 encode the cytoplasmic domain of CD6 (Figure 2). Analysis of single-nucleotide polymorphisms (SNPs) in the *cd6* locus has unveiled association between MS and the SNP rs17824933 in exon 1 [19, 20]. Besides this well-known SNP which is associated with CD6 lower expression [21], another 8 nonsynonymous SNPs characterize the coding region but their effects are unknown.

### 2.2. CD6 Transcripts

Hematopoietic precursors thymocytes, mature T and NK cells, a subset of normal B lymphocytes and most of those from lymphocytic chronic leukemia (CLL) transcribe mRNAs for CD6. In B cells, CD6 expression predominates in mature B cells (usually in association with CD5) which make IgM, but do not isotype switch [22]. CD6 mRNA is also detected in various brain regions, most notably in basal ganglia and cortex cerebellum [23, 24].

Northern blot detects a 3-kb band corresponding to CD6 mRNA in T lymphocytes and brain cells. Its intensity augments after exposure to phorbol myristic acetate (PMA), a protein kinase C activator (PKC), or incubation with phytohaemagglutinin (PHA). Controversy persists over the influence of the B-cell Ag receptor (BCR) engagement on CD6 expression. Some authors, but not all, observed an induction of CD6 upon anti-IgM or superantigen *Staphylococcus aureus* Cowan I stimulation [22, 25].

Among the mRNAs for CD6, several alternative splices have been identified by RT-PCR, and confirmed by sequencing (Figure 2): one lacking exon 5 (CD6ΔD3) in T cells, and at least four isoforms lacking exon(s) for the cytoplasmic domain of CD6: Δ8, Δ8-9, Δ8/Δ12 or Δ9/Δ12 in CLL [26]. An exon 9-lacking splicoform has also been described in mice [27, 28]. CD6ΔD3 expression is upregulated throughout the maturation of thymocytes, and following stimulation with PHA [29]. However, the full-length CD6-encoding cDNA predominate in resting and activated cells.

### 2.3. CD6 Protein

Of the 105 kDa-molecular weight (MW) of glycoprotein, 35 kDa are accounted for by polysaccharides. This heavy glycosylation is rendered possible by eight putative sites for *N*-glycosylation and two putative sites for *O*-glycosylation in the extracellular part of the molecule. In addition, when lymphocytes are activated by PMA or by fetal calf serum, CD6 serine, threonine, and tyrosine (Y) residues are hyperphosphorylated and the MW of the related band increased from 105 to 130 kDa [30]. Comparison of the CD6 SRCR domains between human and mouse shows 54% aa identity for SRCR-D1, 84% for SRCR-D2, and 81% for SRCR-D3 [7], and 69% for the transmembrane domain [28].

The 244-aa cytoplasmic tail of CD6 harbors nine threonine and 32 serine residues. Two proteins, SLP-76 and synthenin-1, may be physically associated with Y662 of human CD6 [31, 32]. Of note, SLP-76 that activate PKC and MAPK pathways requires Y662 phosphorylation to bind CD6 [31, 33]. Synthenin-1 is important in protein trafficking, cell adhesion, and activation of Sox4 and eIF5A TFs. In rat, CD6 associates with different protein tyrosine kinases, including Lck, Fyn, Zap-70, and Itk [34].

In T and B cells, interactions with kinases are reinforced when aa are Y phosphorylated upon PMA or, anti-CD3 stimulation, with, but also without, CD2 or CD4 cross-linking [35, 36]. Ligation of CD6 with anti-CD6 mAb on both normal and leukemic human T cells triggers off the activation of the MAPK cascade with phosphorylation of Erk1/2, p38 and JNK [37]. However, cross-linking the anti-CD6 mAb is required to launch the MAPK pathways.

## 3. Biological Functions

### 3.1. CD6-Positive and CD6-Negative Cells

There are no CD6 knockout or CD6-knockin mice, yet CD6-positive cells and CD6-negative cells were compared in T and B cells. In T cells, CD6-positive lymphocytes proliferate more than CD6-negative lymphocytes in the presence of stimulation by allo-Ag presenting cells (APCs) [38]. T- and B-cell proliferation in response to mitogens is similar in CD6-positive cells and in CD6-negative cells [22, 38]. The latter B cells, in the presence of T cells and PMA, produce high amount of IgM and IgG, whereas CD6-positive B cells produce little IgM, and do not make IgG [22].

### 3.2. Transligation

#### 3.2.1. Main CD6 Ligand

ALCAM belongs to the immunoglobulin (Ig) cell adhesion molecule superfamily. It is expressed on bone marrow (BM) stromal cells, thymic epithelial cells, activated T and B cells, monocytes, dendritic cells, synovial fibroblasts, keratinocytes, and mesangial stem cells. ALCAM was identified as a ligand of CD6 [39, 40]. ALCAM expression is tightly regulated, and indeed mitogen-activated peripheral blood mononuclear cells (PBMCs) express ALCAM for 24 to 48 h, but not thereafter [41]. 

The extracellular region of ALCAM consists of five extracellular Ig domains (D1–D5). The two NH2-terminal domains are related to variable (V)-type and the three proximal domains to constant (C)-type Ig domains. The *N*-terminal domain of ALCAM interacts with the conserved SRCR-D3 of CD6, while the C-proximal domains oligomerize ALCAM. The CD6 binding domain for ALCAM is highly conserved, explaining the cross-species CD6/ALCAM interaction between humans, mice, and chickens. This is also the reason why it is so difficult to mount an Ab response against the CD6 binding site [41]. Like most other leukocyte membrane interactions, the heterotypic ALCAM-CD6 interaction is of low affinity, albeit 10- to 100-fold higher than the homotypic ALCAM-ALCAM interaction [42]. Binding studies with CD6 fusion proteins has revealed that CD6-SRCR-D2 (exon 4) and CD6 spacer domain (exon 6) are important to stabilize the interaction between CD6-SRCR-D3 and ALCAM [43]. Mutagenesis analysis has shown that three residues in the *C*-terminal region of CD6-SRCR-D3 and nine residues in the ALCAM-D1 domain are involved in the binding/interaction CD6/ALCAM [44]. 

The binding of CD6 to ALCAM is dominant for CD34^+^ hematopoietic progenitor binding to BM stromal cells [45], thymocyte binding to thymic epithelial cells [39, 40], and T-cell-dependent proliferation [46]. In pathological settings, supplemental functions have been ascribed to the complex CD6/ALCAM. First, CD4^+^-T-cell transmigration into the central nervous system is related to CD6/ALCAM interaction at the border between blood and brain [47]. Second, ALCAM overexpression entrances the lymphocyte recruitment to inflammatory sites, including RA synovium, SS exocrine glands, MS brain, and microenvironment of selected cancers [47–50].

#### 3.2.2. Accessory Ligands for CD6

The list of CD6 ligands is extremely long, based on the observation that CD6 fusion protein immunoprecipitate from a human epithelial cell live not only with ALCAM, but also with a 90-kDa and a 45-kDa proteins [51]. Another clue to additional ligand(s) for CD6 is that the binding of T cells to epithelial cells cannot be totally blocked by anti-CD166 Ab and by anti-CD6 SRCR-D3 Ab [52]. 

Following activation with IFN-*γ* a 130-kDa protein designated 3A11 arises on synovial fibroblasts, thymic fibroblast, and keratinocytes [52–54]. The interaction could be abrogated by a CD6 fusion protein or an anti-CD6 SRCR-D1 Ab suggesting that CD6 domain SRCR-D1 recognizes alternative ligands.

#### 3.2.3. Pathogen Recognition

The Lozano's group has recently reported on the binding of CD6 and CD5 to pathogen-associated molecular patterns [55, 56]. Catching of pathogen by CD6 is dose dependent, saturable, facilitated by calcium, and achieved by the three SRCR domains. Microbes induce signaling with MAPK/Erk activation and cytokine release. This Toll-like receptor-independent process requires the integrity of the cytoplasmic domain as demonstrated with CD5. Interestingly, soluble CD6 and CD5 have been detected in normal human sera, and their levels found to be increased in autoimmune diseases, thus suggesting a feedback loop to reduce direct lymphocyte activation by pathogens [57, 58].

### 3.3. Cis Ligation

#### 3.3.1. CD6-CD3 Interactions and Immunological Synapse

The junction between a T lymphocyte and an APC is designated as immunological synapse (IS) and assigned to an active mechanism. First, T-cell Ag receptor (TCR) ligands and costimulatory molecules are engaged in an external ring of the nascent IS, translocated into the central cluster, and fixed in the heart of the IS. Finally, the IS consists of the central supramolecular activation cluster (cSMAC) enriched in TCR/CD3 complexes, CD4/CD8/CD2/CD5 costimulatory molecules, and kinases (PKC, Lck and Fyn), and surrounded by a supramolecular activation clusters (pSMAC) including LFA-1 and talin.

The fact that CD6 exists both in pSMAC and in cSMAC suggests the possibility of two functions, each being related to its location [29, 59, 60]. *At the pSMAC*, CD6 binds to ALCAM. This is stable, long lasting and involved in the contact initiation between T cells and APCs. Then blocking CD6-ALCAM interaction reduces significantly T-cell/APC contact and impairs the T-cell proliferation. This could be achieved with anti-CD6-SRCR-D3 Ab or ALCAM-blocking Ab. In addition, when the cells express an alternative isoform of CD6, CD6ΔD3, devoid of ALCAM binding domain, the contact of lymphocytes with APC is lost [29]. *At the cSMAC*, CD6 is physically associated with the TCR/CD3 complex, and, as such, participates in the proliferation of T cells. Since T1h related anti-CD6-SRCR-D1 Ab are able to inhibit T-cell proliferation without affecting T-cell/APC contact it could be postulated that this interaction involved the SRCR-D1 part of CD6.

#### 3.3.2. CD6 and CD5 Association

Confocal microscopy, coupled with the FRET technology [61], establishes that 12% of the CD6 molecules are associated with CD5 molecules in resting T cells. Upon activation, this association is reinforced and the CD6-CD5 complexes migrate to the IS where they colocalize with TCR/CD3 within the cSMAC. Of note, complexes of cross-linked CD5 and CD6 are internalized, and, by doing so, confirm their physical link.

Interactions between CD6 and CD5 proceed through their extracellular domains given that, despite truncation of its intracellular tail, CD5 keeps its ability to bind CD6. Which CD6 extracellular domain is involved is currently unknown. The CD5-CD6 interaction is not unique, since CD5 SRCR-D2 interact with the BCR [62], CD5 SRCR-D3 interferes with itself [63] and interacts with CD2 [64]. Interestingly, when CD6 and CD5 are physically associated, CD6 is phosphorylated on its Y629 and CD5 on its Y429, suggesting that their association contributes to TCR- or BCR-independent activation. This was demonstrated in rat T cells by immunoprecipitation where CD5 was associated with hyperphosphorylated 130-kDa CD6, and CD6 with hyperphosphorylated CD5 [65]. 

Over the thymic maturation, the expression CD6, that of CD5 (and thereby their interactions) are tightly regulated, and contributes to the thymocyte selection. Immature double-negative CD4^−^CD8^−^ thymocytes express CD6 and low levels of CD5, at immature double-positive CD4^+^CD8^+^ stage CD6 expression is stable and CD5 is increased, finally at single-positive CD4^+^ or CD8^+^ stages both molecules are highly expressed [66, 67]. When compared to single-positive thymocytes, cell surface expression on resting peripheral blood T cells is lower for CD6 and similar for CD5. 

Within the B lymphocytes, the CD6-expressing cells overlap, but not entirely, with the CD5-expressing cells [68]. CD5-expressing B cell, designated B1 cells, as opposed to conventional CD5-nonexpressing B2 cells, produce polyspecific Abs and, therefore, constitute the main source of natural Abs. In fact, the CD6 molecule acts as a positive regulator of the BCR while CD5 is a negative regulator.

## 4. Anti-CD6 Immunotherapy

### 4.1. Anti-CD6 Monoclonal Abs

**Figure 3:**
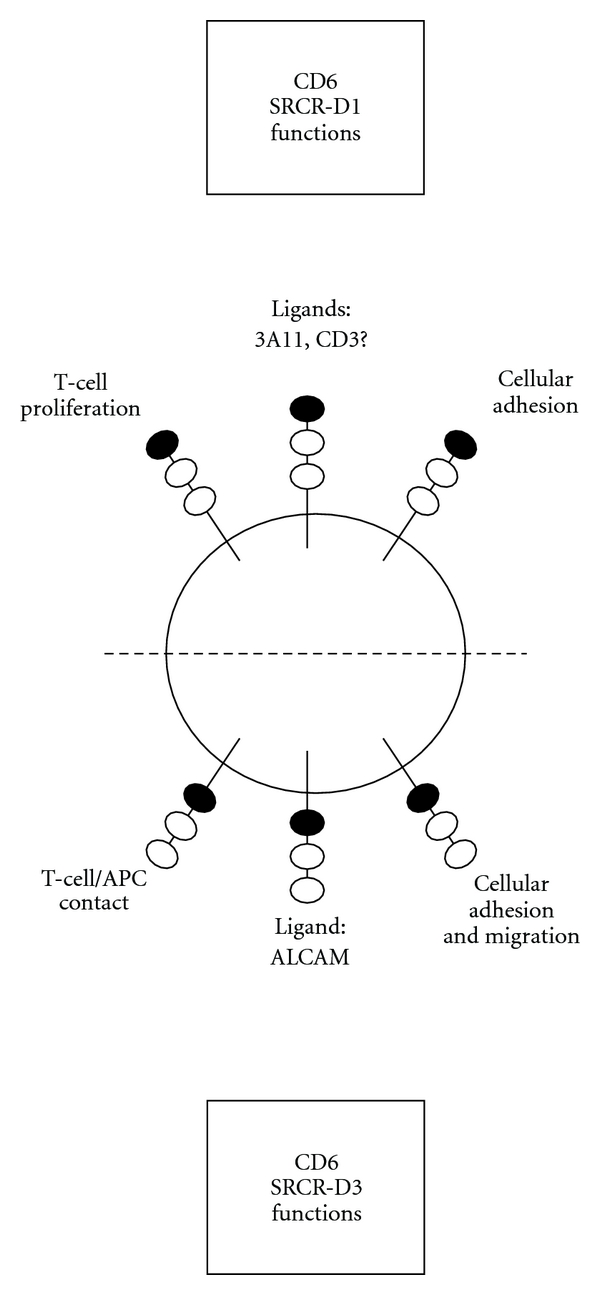
CD6 functions according to the CD6 scavenger receptor cystein-rich (SRCR) domain involved.

Different anti-CD6 mAbs have been raised, mainly against the first and third SRCR domains of CD6. Four main epitopes are described in the SRCR-D1 [69]. Briefly, anti-CD6-SRCR-D1 mAbs hinder autoreactive and nonautoreactive T-cell proliferation while anti-CD6-SRCR-D3 mAb prevents the adhesion and Ag-dependent activation of lymphocytes via its interaction with ALCAM (Table 2 and Figure 3). 

Ligation of the CD6 cell surface molecule by cross-linking an anti-SRCR-D1 mAb mimics the CD6-CD166 interaction and delivers a direct signal by activating MAPK [37, 55]. T-cell proliferation could be achieved or amplified when ligation of the CD6 molecule is associated with a second signal mediated by the ligation of CD3, CD28, or with PMA [69]. This proliferation is IL-2 dependent.

In B cells, CD6 ligation was not found to induce the proliferative response of CLL but to protect from anti-IgM-mediated apoptosis through bcl-2 induction [25]. CLL B-cell spontaneous apoptosis is not affected by CD6 cross-linking.

### 4.2. Anti-CD6 and Clinical Studies

The murine IgM anti-T12/CD6 mAb has been used experimentally *in vivo* as an anti-T-cell agent in MS, leukaemia, in acute graft-versus-host GvH disease, and in acute renal transplant rejection [3, 4, 72]. However, circulating CD6-positive T cells disappear completely the first week, the appearance of blocking human anti-mouse IgM in the serum by 78% of the patients, seven days after infusion, together with allergic reactions limits the efficiency of the mAb. In addition, the anti-T12/CD6 mAb associated with rabbit complement was also used *ex vivo* for removing T cells from the donor BM, as an attempt to reduce the incidence of acute and chronic GvH disease. These allogeneic CD6-depleted BM transplantations have been used successfully in patients with hematologic malignancies and severe combined immunodeficiency [73, 74]. After BM transplantation, NK cells are reconstituted early, and T cells are detected after four to eight weeks including T-cell functional abnormalities and a significant fraction of CD6-nonexpressing T cells [75]. A decade ago, transplantation of anti-T12/CD6 T-cell-depleted BM was supplanted by the selection of stem cells CD34^+^.

CD6 mAb ior T1 (IgG2a,k) was raised in BALB/c mice immunized with PBMCs from a patient with Sezary's syndrome [6, 7]. Ior T1 mAb, another anti-CD6 SRCR-D1 mAb, has been used with success in cutaneous T-cell lymphoma and in a patient with psoriasis [1]. A technetium-99m-labeled ior T1 mAb was tested in a phase II clinical trial in RA demonstrating a link between the concentration used and the clinical improvement [2].

### 4.3. Humanized Anti-CD6 Monoclonal Ab

To reduce its immunogenicity, ior T1 mAb has been genetically engineered to progressively replace murine content with the human counterparts. In the first step, a chimeric human-mouse IgG was generated by exchanging corresponding heavy chain C domains between the human IgG1 and the murine IgG2a. In the second step, the two homologous mouse and human V region sequences were compared and Ag sequences of T cells were analyzed. In the third step, 11 aa present in the V region were subjected to point mutations to make them human and/or to modify amphiphatic motifs [6]. The resulting recombinant Ab (T1h) retained its Ag-binding affinity and was less immunogenic than the murine original [6, 7]. Humanized anti-CD6 T1h recognizes a conformational epitope independent of the CD6 *N*-glycosylation of SRCR-D1. As suspected, it does not block CD6-ALCAM interactions suggesting that T1h could not inhibit ALCAM-related T-cell activation and prevent T-cell migration.

## 5. Conclusions

CD6 SRCR-D1 contributes to lymphocyte proliferation, adhesion and survival process. Indeed, the humanized anti-CD6 SRCR-D1 clone T1h which does not interfere with the CD6-ALCAM interactions may have therapeutic implications in oncology, transplantation, and autoimmunity. The recent data obtained with soluble recombinant CD6 in an experimental model of septic stock support this model.

## Figures and Tables

**Table 1:** Anti-CD6 and clinical studies.

Clone	Diseases	Effect	Author
Ior T1	T cell lymphoma, Psoriasis	Clinical improvement	[1]
Ior T1-99Tcm	Rheumatoid arthritis	Clinical improvement	[2]
T12	Multiple sclerosis	T cell depletion	[3]
T12	BM transplantation in SCID patient	Prevent acute GvH disease	[4]
T12	Allogeneic BM transplantation	T cell elimination	[5]

GvH disease: graft-versus-host disease; BM: bone marrow, IV: intravenous; SCID: severe combined immunodeficiency.

**Table 2:** Anti-CD6 SRCR-D1 mAbs and functional effects.

Clone	Functional response	Cross-link	Author
BLTP6a, UMCD6 and MT502	Block T-cell Ag dependant activation	No	[18]
161.8	Induces Erk1/2 activation	Yes	[37]
IorT1, 2H1, T12, 6D3, Dako-CD6	Enhance anti-CD3, anti-CD28 and PMA T cell proliferation	Yes	[70, 71]
Ior T1	Protects B-CLL from anti-IgM apoptosis	Yes	[25]
UMCD6	Blocks T-cell/keratinocyte interaction	No	[18]
